# Safety and tolerability of intravitreal umedaptanib pegol (anti-FGF2) for neovascular age-related macular degeneration (nAMD): a phase 1, open-label study

**DOI:** 10.1038/s41433-023-02849-6

**Published:** 2023-12-01

**Authors:** Daniel S. Pereira, Kazumasa Akita, Robert B. Bhisitkul, Toshiaki Nishihata, Yusuf Ali, Emiko Nakamura, Yoshikazu Nakamura

**Affiliations:** 1 RIBOMIC USA Inc, Berkeley, CA USA; 2 RIBOMIC Inc., Minato-ku, Tokyo Japan; 3 Department of Ophthalmology, University of California San Francisco, San Francisco, CA USA; 4 Institute of Medical Science, The University of Tokyo, Minato-ku, Tokyo Japan

**Keywords:** Drug discovery, Medical research

## Abstract

**Objective:**

To evaluate the efficacy and safety of a single-dose intravitreal umedaptanib pegol (anti-FGF2, investigational new drug) for the treatment of neovascular age-related macular degeneration (nAMD).

**Methods:**

Nine participants who had a diagnosis of refractory nAMD were enrolled and received a single intravitreal injection of umedaptanib pegol at increasing doses of 0.2, 1.0 or 2.0 mg in the study eye.

**Results:**

All three doses of umedaptanib pegol evaluated in the study were safe and well tolerated. No severe adverse event (AE) was observed in the study. There was an improvement in retinal fluid measured by central subfield thickness (CST) in most subjects. Remarkably, all three subjects who received 2.0 mg/eye showed improvement of more than 150 μm.

**Conclusions:**

Intravitreal umedaptanib pegol was safe, well tolerated, and demonstrated an indication of bioactivity in participants that have persistent subretinal fluid refractory to the treatment with anti-VEGFs.

## Introduction

**Fig. 1:**
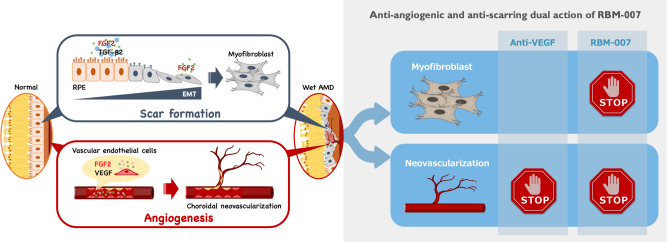
Schematic model of the dual role of FGF2 in angiogenesis and fibrotic scar formation in the retina. Upon inflammation, FGF2 in the presence of TGFβ2 stimulates RPE cells to undergo EMT to fibroblasts, leading to scar formation. In parallel, FGF2 and VEGF act as vascular endothelial cell mitogens in the initiation and maturation of angiogenic vessels. Hence, FGF2 should be a new therapeutic target in nAMD by its dual action.

Age-related macular degeneration (AMD) is the leading cause of visual loss in the elderly population [1]. The number of people with AMD was estimated to be 196 million in 2020 and will reach 288 million in 2040 globally [2]. The loss of central vision in neovascular age-related macular degeneration (nAMD) is caused by choroidal neovascularization (CNV), resulting in macular haemorrhage, effusion, and fibrosis [3] (Fig. 1).

The current standard therapy for nAMD is to target vascular endothelial growth factor (VEGF) using ranibizumab (Lucentis^®^, Roche/Genentech), aflibercept (Eylea^®^, Regeneron Pharmaceuticals), bevacizumab (Avastin^®^, Roche/Genentech) and faricimab (Vabysmo^®^, bispecific anti-VEGF/anti-Ang2 drug, Roche/Genentech) [4, 5]. Frequent intravitreal injections of anti-VEGF drugs have been shown to be associated with major visual benefits in participants with AMD [6–8]. Despite their present efficacy, anti-VEGF agents also have several limitations. Participants may require a high injection frequency during years of treatment, leading to a high treatment burden. In addition, compared with participants in clinical trials, real-world participants showed worse visual outcomes, possibly due to poor compliance [9, 10]. Moreover, the risk of developing retinal scarring and geographic atrophy was increased after 2–5 years of treatment [11].

Recent studies have shed light on the role of fibroblast growth factor-2 (FGF2) in disease progression of nAMD. In mammals, FGFs have 22 known members that exert important functions in regulating cell proliferation, differentiation, and migration [12, 13]. Upon binding to tyrosine kinase FGF receptors, FGFR1-FGFR4, FGFs activate essential signalling pathways, such as the mitogen-activated protein kinase (MAPK)/ERK and JNK pathways, that are centrally involved in angiogenesis, tissue remodelling, and regeneration, including repair of neuronal damage, skin wound healing, joint protection, and control of hypertension. FGF2 is a major member of the FGF family along with FGF1 and has been implicated in the pathophysiology of both angiogenesis and fibrosis [14, 15]. It has been demonstrated that FGF2 stimulates the growth of vascular endothelial cells and tubular structure formation [16] in addition to promoting VEGF expression [17, 18]. The angiogenic activity of FGF2 was reportedly stronger than that of VEGF in a mouse corneal micropocket assay [19, 20]. Moreover, FGF2 in combination with TGFβ2 stimulates epithelial-mesenchymal transformation (EMT) in retinal pigment epithelial (RPE) cells [21], which might contribute to submacular fibrosis.

To address the targetability of FGF2 in nAMD treatment, the anti-FGF2 aptamer, umedaptanib pegol (formerly called RBM-007 [22]), was examined for the treatment of named in animal models. Umedaptanib pegol is composed of 37 nucleotides, whose ribose 2′ positions are modified to resist ribonucleases, in addition to being 5′-PEGylated and 3′-conjugated with an inverted dT to confer an advantageous pharmacokinetic profile [22]. Umedaptanib pegol binds strongly and specifically to FGF2 with the dissociation constant of 2 pM and blocks the interaction between human FGF2 and its receptors FGFR1-FGFR4 [21, 22]. In the in vivo studies conducted in mice and rats, umedaptanib pegol was able to inhibit FGF2-induced angiogenesis, laser-induced CNV, and CNV with fibrosis [21]. Pharmacokinetic studies of umedaptanib pegol in the rabbit vitreous revealed high and relatively long-lasting profiles [21]. Moreover, combined treatment with umedaptanib pegol and ranibizumab showed a synergistic effect in preventing CNV [21]. This therapeutic potential is further supported by the finding that FGF receptor double-conditional knockout (*Fgfr1/2*) mice showed a marked reduction in CNV accompanied by a decrease in the level of FGF2 upon laser injury [23]. Additionally, FGF2 was the only essential ligand in the in vivo models of CNV, in keeping with FGF2 regulation of pathogenic angiogenesis via the STAT3 pathway [24]. The anti-angiogenic and anti-scarring dual action of umedaptanib pegol holds promise as an additive or alternative therapy to anti-VEGF treatments for nAMD (see Fig. 1).

We report on our clinical trials first designed as phase 1 (SUSHI), open-label, safety and feasibility study of a single-dose umedaptanib pegol in subjects with refractory nAMD, and then followed by phase 2 trials in the accompanying manuscript.

## Methods

### Investigational new drug, umedaptanib pegol

The drug substance for umedaptanib pegol intravitreal injection is a sodium salt of a single-stranded oligonucleotide aptamer with thirty-seven structure-forming nucleotides in length that terminates at the 3’-end in an inverted 2’-deoxy-thymidine and at the 5’-end in an aminohexyl linker. The aminohexyl linker is covalently conjugated via an N-alkyl amide linkage with one branched 2 × 20-kDa mono-methoxy polyethylene glycol (PEG) unit. The chemical formula of umedaptanib pegol is as follows: RNA, amC6-5’-(Guo_m_-Guo_m_-Guo_m_-Ado_m_-Uro_m_-Ado_m_-Cyd_m_-(2’-deoxy-2’-fluoro)Uro-Ado_m_-Guo_m_-Guo_m_-Guo-Cyd_m_-Ado_m_-Uro_m_-(2’-deoxy-2’-fluoro)Uro-Ado_m_-Ado_m_-Uro_m_-Guo_m_-(2’-deoxy-2’-fluoro)Uro-Uro_m_-Ado_m_-Cyd_m_-Cyd_m_-Ado_m_-Guo-(2’-deoxy-2’-fluoro)Uro-Guo-(2’-deoxy-2’-fluoro)Uro-Ado_m_-Guo_m_-Uro_m_-Cyd_m_-Cyd_m_-Cyd_m_)-3’-3’-(2’-deoxy)Thy-5’; 5’-ester with 2,3-Bis [methoxy-poly(oxy-ethylene)]-1-carbamyl 7-aza-hexylene-oxy phosphate, sodium salt. Where Ado = adenylyl; Cyd = cytidylyl; Guo = guanylyl; Uro = uridylyl; Thy = thymidinyl; amC6 = aminohexyl; Nnn_m_ = 2’-methoxy-nucleotide. Umedaptanib pegol -007 injectable solution is a single-use, preservative-free, sterile solution formulated in 3.6% or 4.9% mannitol vehicle for intravitreal administration.

### Phase 1 (SUSHI) study design and participants

The SUSHI study (www.clinicaltrials.gov, identifier, NCT03633084) enrolled eligible subjects aged 55 years or older and had been diagnosed with nAMD in the eye under study, for which previous standard treatment with intravitreal anti-VEGF agents (aflibercept, bevacizumab or ranibizumab) has recently demonstrated incomplete resolution of exudation, as assessed by spectral domain optical coherence tomography (SD-OCT). Other inclusion criteria were BCVA of 65–10 letters (≤20/50 and ≥20/640 Snellen vision equivalent), presence of macular oedema or subretinal fluid on SD-OCT, choroidal neovascular lesions ≤9-disc areas (DA) and lesion composed of ≤50% subretinal haemorrhage.

**Fig. 2:**
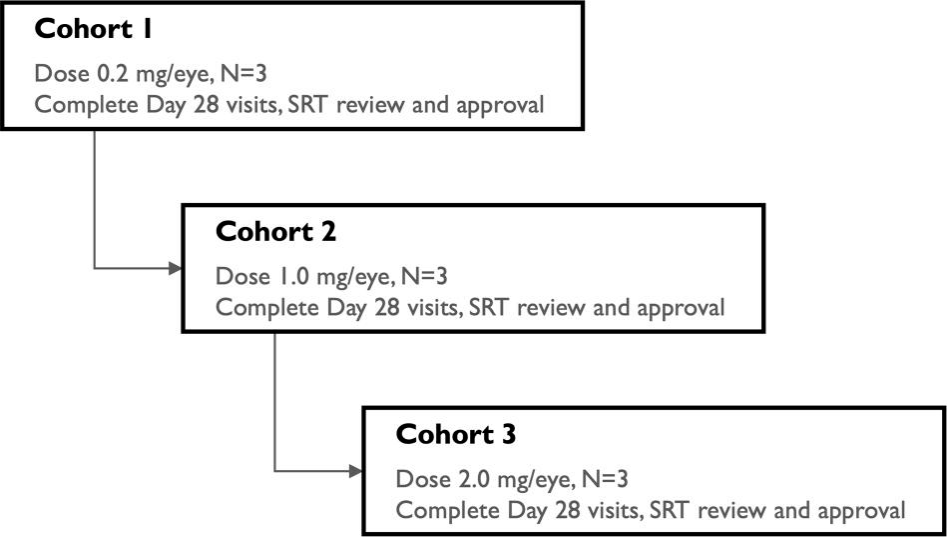
Flowchart showing the dosing regimen for subjects enrolled in the study. Cohort 1 subjects were pretreated with aflibercept, cohort 2 subjects with bevacizumab and ranibizumab, and cohort 3 subjects with aflibercept, ranibizumab and bevacizumab.

The study was conducted at four study sites in the United States between August 2018 and June 2019. Nine participants who had a diagnosis of refractory nAMD were enrolled. Following a screening evaluation, subjects received a single intravitreal injection of umedaptanib pegol at increasing doses of 0.2, 1.0 or 2.0 mg in the eye under study and no additional doses were administered thereafter (Fig. 2). The primary endpoint of the study was at 28 days post-injection of umedaptanib pegol, with safety evaluation through 56 days. The study was initiated with the lowest dose of 0.2 mg in the first cohort of three subjects, proceeding to a second cohort of three subjects at a dose of 1.0 mg, then a third cohort of three subjects at a dose of 2.0 mg. Decisions regarding proceeding to each sequential cohort were based on the recommendations of the Safety Review Team (SRT), consisting of external Retina Specialists and the Medical Monitor (Fig. 2). The first subject of each dose cohort was assessed by the SRT at 7 days after injection of umedaptanib pegol to determine if safety was acceptable. Umedaptanib pegol treatment was then given to the remaining two subjects in that dose cohort. Upon completion of the primary endpoint at 28 days post-umedaptanib pegol injection by all three subjects in the cohort, the SRT reviewed the safety of these subjects. The next dose cohort was then initiated, and the same schedule of interval SRT evaluations was repeated for that cohort.

#### Clinical study sites

The SUSHI study was conducted in the following clinical sites: Retinal Consultants Medical Group, Sacramento, CA (PI: Joel Pearlman), Retinal Consultants Medical Group, Sacramento, CA (PI: Margaret Chang), Stanford University, Stanford, CA (PI: Diana Do), and Bay Area Retina Associates, Walnut Creek, CA (PI: Subhransu Ray).

### Drug administration procedure

Umedaptanib pegol for intravitreal injection is formulated in a proprietary, clear, aqueous solution. Intravitreal injections were given according to standard of care (SoC) techniques used in modern retinal practice. Briefly, a sterile lid speculum was placed, and local/topical anaesthesia was administered. The conjunctiva and ocular adnexa were prepared with povidone-iodine. A 30-gauge needle was used for all injections, which were given 4.0 mm from the limbus. In this SUSHI study, intraocular pressure (IOP) was measured 30 (±10) min after the intravitreal injection, if ≥10 mmHg from pre-injection IOP, it would be re-evaluated in 60 (+10) min. If still ≥10 mmHg compared to pre-injection IOP, the subject was prescribed a topical IOP-lowering medication until the return for follow-up per the clinical investigator’s discretion, and the IOP increase of ≥10 mmHg was reported as an adverse event (AE).

### Ethics statement

The SUSHI studies were conducted at four study sites in compliance with the Declaration of Helsinki, US Code 21 of Federal Regulations, and the Harmonized Tripartite Guidelines for Good Clinical Practice (1996); and were reviewed and approved by the appropriate Ethics Committees or institutional review boards. Informed consent was obtained from all study participants.

## Results

### Study design

The SUSHI study is a multicentre, open-label, dose-escalating study assessing the safety, tolerability, and bioactivity of a single intravitreal injection of umedaptanib pegol in nine adult patients between 71 and 92 years old (five females and four males, Caucasian) with nAMD refractory to treatment with anti-VEGF medications. Following a screening evaluation, subjects received a single intravitreal injection of umedaptanib pegol at a dose of 0.2 (cohort 1), or 1.0 (cohort 2) or 2.0 (cohort 3) mg in the eye tested (Fig. 2). There were three patients in each cohort.

### Safety and tolerability

All three doses of umedaptanib pegol (0.2, 1.0, and 2.0 mg) evaluated in the study were safe and well tolerated. No severe AE was observed in the study. There was a single drug-related AE (mild iritis) that was resolved within one day by administration of topical corticosteroid drops. In addition, there were two cases of subconjunctival haemorrhage, which were related to the intravitreal injection procedure.

### Efficacy

**Fig. 3:**
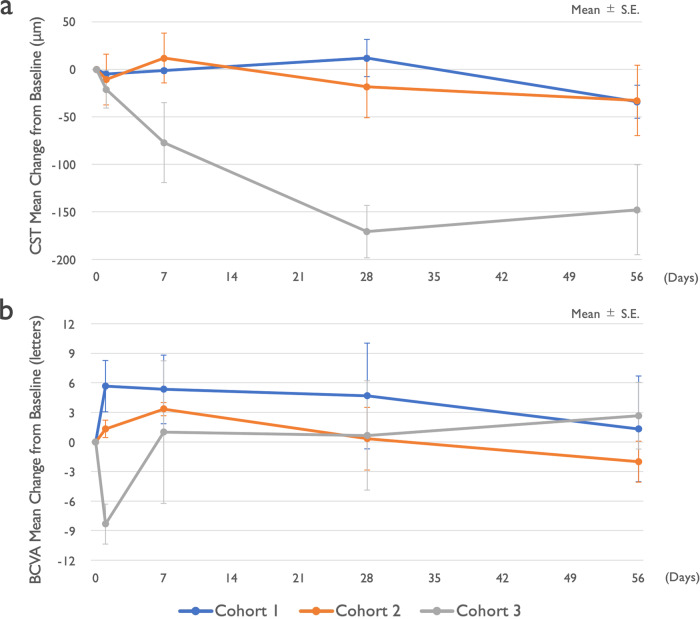
Mean change of vision and macular anatomy in the SUSHI study. Nine refractory nAMD patients received a single intravitreal injection of umedaptanib pegol at doses of 0.2 (cohort 1), 1.0 (cohort 2) or 2.0 mg (cohort 3) in the eye under study. **a** Change of central subfield thickness (CST) monitored by OCT. **b** Change of best-corrected visual acuity (BCVA) monitored by EDTRS letters. Error bars represent standard error.

**Fig. 4:**
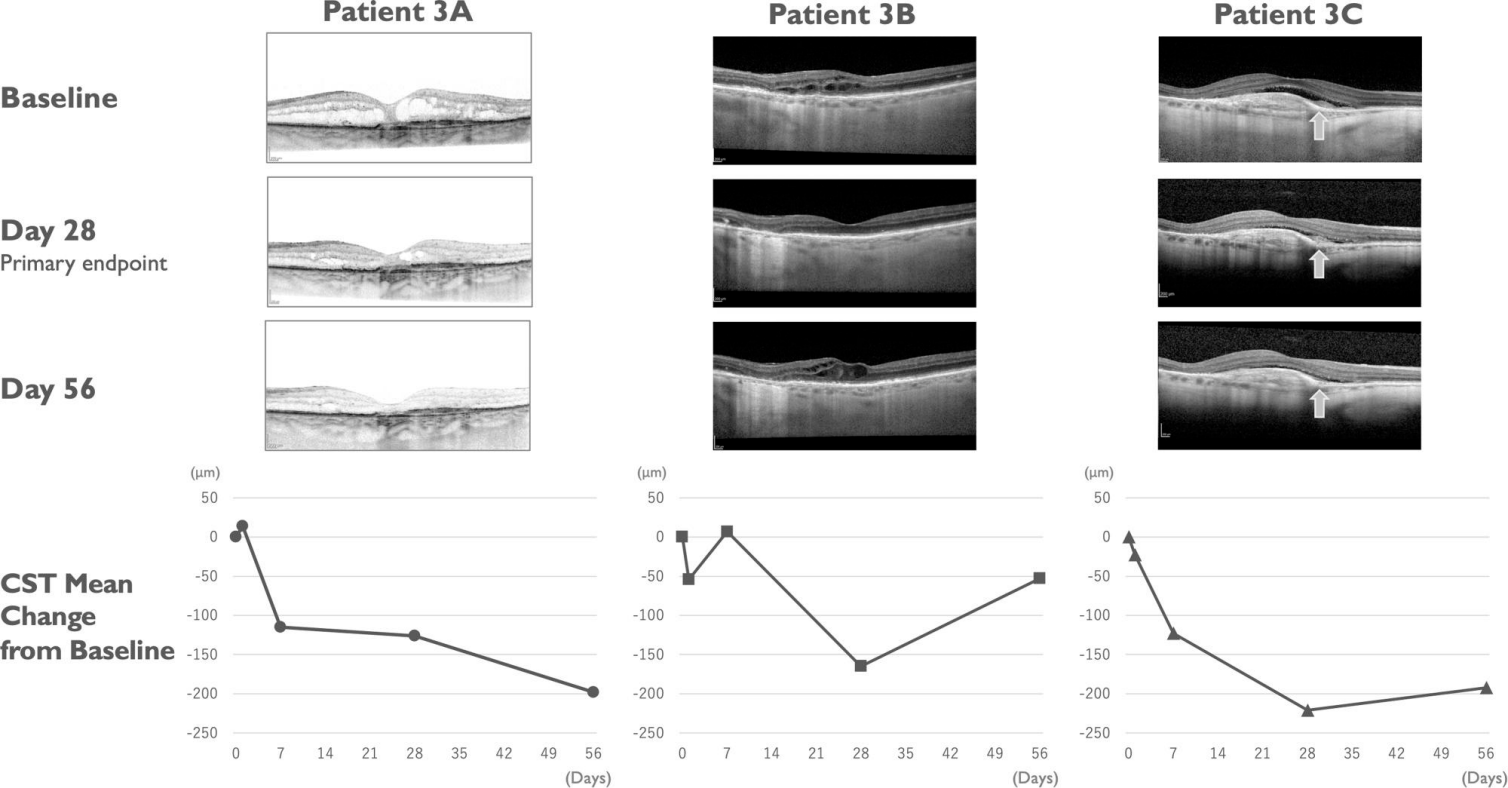
Change of macular anatomy in individual patients of cohort 3 in the SUSHI study. Case examples showing reduction in retinal fluid in cohort 3 patients. SD-OCT images at baseline (top), day 28 (primary endpoint, middle) and day 56 (bottom) are shown. Each patient CST mean change from baseline is presented under the SD-OCT image. Patient 3A, 86-year-old female (Caucasian) who received 58 injections of anti-VEGFs for 9 years before this study; Patient 3B, 92-year-old female Caucasian) who received 19 injections of anti-VEGFs for 3 years before this study; Patient 3C, 86-year-old female (Caucasian) who received 5 injections of anti-VEGFs for 1 year before this study. SHRM is marked by arrows in subject 3.

There was an improvement in retinal fluid quantities measured by central subfield thickness (CST) in 6/9 subjects at day 28 and in 7/9 subjects at day 56 (Fig. 3a). All three subjects in cohort 3 (2.0 mg/eye) showed improvement of more than 150 μm at day 28 and/or day 56 (Fig. 3a). Individual optic coherence tomography (OCT) images of cohort 3 are shown in Fig. 4. Improvement of best-corrected visual acuity (BCVA) over baseline at day 28 was observed in 5/9 subjects (Fig. 3b). Based on these results, there is an indication of bioactivity of umedaptanib pegol in patients that have persistent subretinal fluid refractory to the treatment with anti-VEGFs. Furthermore, based on the OCT data, umedaptanib pegol administration led to reabsorption of subretinal hyperreflective material (SHRM), which is a significant risk factor for scar formation, in patient 3C of cohort 3 (Fig. 4, right) [25]. Resolution of SHRM has been observed during anti-VEGF monotherapy or combination therapy in treatment-naïve nAMD patients [26, 27].

### Blood PK profile

There was small or negligible (below quantitation limit) exposure of umedaptanib pegol in plasma. No drug was detected in plasma in any subjects in cohort 1 at day 28. The highest concentration of drug detected at day 28 was 17.5 ng/mL in one subject in cohort 3. The highest concentration of drug detected in any cohort during the trial was 269 ng/mL in one subject at day 1 in cohort 3, demonstrating that the systemic exposure of umedaptanib pegol is quite low when administered via the intravitreal route.

## Discussion

A significant unmet need exists with anti-VEGF monotherapy regardless of the benefit in anti-VEGF medications in patients with nAMD [8, 28–31]. Numerous clinical trials have been completed or are underway to fill the remaining unmet needs of the anti-VEGFs.

In the present investigation, escalating intravitreal doses of an anti-FGF2 pegylated-aptamer umedaptanib pegol were administered in refractory nAMD patients. This is the first in human clinical trial to target FGF2 via intravitreal administration. Accordingly, umedaptanib pegol was given at increasing doses at 0.2, 1.0, and 2.0 mg/eye. Significantly, no unexpected local or systemic adverse safety signals were observed after the administration of the investigational drug.

The participants in this study had long histories of intravitreal anti-VEGF therapy and were considered refractory to these treatments. It was remarkable that there appeared to be an improvement in retinal fluid levels as measured by central subfield thickness (CST) in most subjects. This was associated with vision improvement in a significant number of treated eyes. The improvement in visual acuity may have been limited by the long duration of CNV disease activity which may have resulted in structural damage to the fovea. These findings suggest that intravitreal umedaptanib pegol is safe and has potential in the treatment of eyes with refractory nAMD where the chorio-retina is no longer responsive to anti-VEGF drugs. This finding supports a distinct mode of action of umedaptanib pegol compared with anti-VEGFs.

Having established the safety of intravitreal umedaptanib pegol, and established a proof of concept, further studies are required to determine the efficacy in the treatment of nAMD. Phase 2 studies are underway.

## Summary

### What was known before

- Intravitreal anti-VEGF drugs (e.g., bevacizumab, ranibizumab, and aflibercept) have become the standard treatment for nAMD.
- As far as is known, VEGF is the only effective target molecule for nAMD monotherapy.
- Participants may require a high injection frequency over years of treatment, leading to a high treatment burden or several complications.
- The real-world studies showed worse visual outcomes, possibly due to poor compliance.

### What this study adds

- Intravitreal umedaptanib pegol was safe, well tolerated, and demonstrated bioactivity in refractory nAMD patients.
- To the best of our knowledge, this is the first report to demonstrate efficacy in monotherapy of nAMD with targets other than VEGF.

## Data Availability

The datasets used and/or analysed during the current study are available from the corresponding author on reasonable request.
